# Editorial: Women and men in physical activity

**DOI:** 10.3389/fphys.2022.988839

**Published:** 2022-08-15

**Authors:** Alessandra Modesti, Simone Luti, Gabriella Pinto, Cristina Vassalle, Pantelis Theodoros Nikolaidis

**Affiliations:** ^1^ Department of Biomedical Experimental and Clinical Sciences “Mario Serio”, University of Florence, Florence, Italy; ^2^ Department of Chemical Sciences, Polytechnic and Basic Sciences School, University of Naples Federico II, Naples, Italy; ^3^ Fondazione CNR-Regione Toscana Gabriele Monasterio, Pisa, Italy; ^4^ School of Health and Caring Sciences, University of West Attica Athens, Athens, Greece

**Keywords:** physical activity, exercice, cardiorespirarory fitness, gender, pathophysiology

Physical activity represents a well-known approach for the prevention and treatment of cardio-metabolic diseases (Sarzynski et al., 2022). In fact, several metabolic changes occur in the organism during exercise, leading to the activation of adaptive mechanisms. These mechanisms aim to establish a new dynamic equilibrium especially at the metabolic level, which enhances health and optimize performance in elite athletes. However, exercise can have negative effects on health, e.g., on inflammatory and redox states (Casuso and Huertas, 2021). Excessive training and effort could lead to chronic fatigue, muscle damage and lower performance. Therefore, final beneficial/adverse exercise consequences are the result of the fine balance between the oxidative stress/inflammation induced by exercise that increases performance and health (hormesis), and the oxidative stress due to excessive effort that causes fatigue and muscle damage (overtraining). Clearly, many variables (e.g., exercise mode, frequency, duration and intensity) may affect the results.

Moreover, women and men exhibit many gender-specific anthropometric and physiologic characteristics, which may influence these adaptive mechanisms and therefore, performance (Santisteban et al., 2022). However, this aspect in particular has often been overlooked. For example, it is known that in long-term endurance exercise, women perform similarly or slightly better than men while in quick bursts with great power no, suggesting a distinct gender dimorphism in metabolism. What molecular mechanisms are activated in man and woman in response to physical exercise? And in recovery, do they act similarly? What about the gender dimorphism in terms of proteomics, lipidomics and metabolomics? What benefits arise from physical activity as an expression and activation of molecular mechanisms? Unfortunately, till now, women are still under-represented compared to men in sport and exercise research, therefore, a comparison to highlight the gender specific adaptation to physical exercise is difficult to do. Generally, data on sex hormonal status are lacking. Instead, hormonal signalling together with muscle fiber type composition and oxidative stress are very important features that characterize gender response in sports (Handelsman et al., 2018). Thus, more studies are needed to fully understand the gender specific physiology and metabolism response during exercise.

**FIGURE 1:**
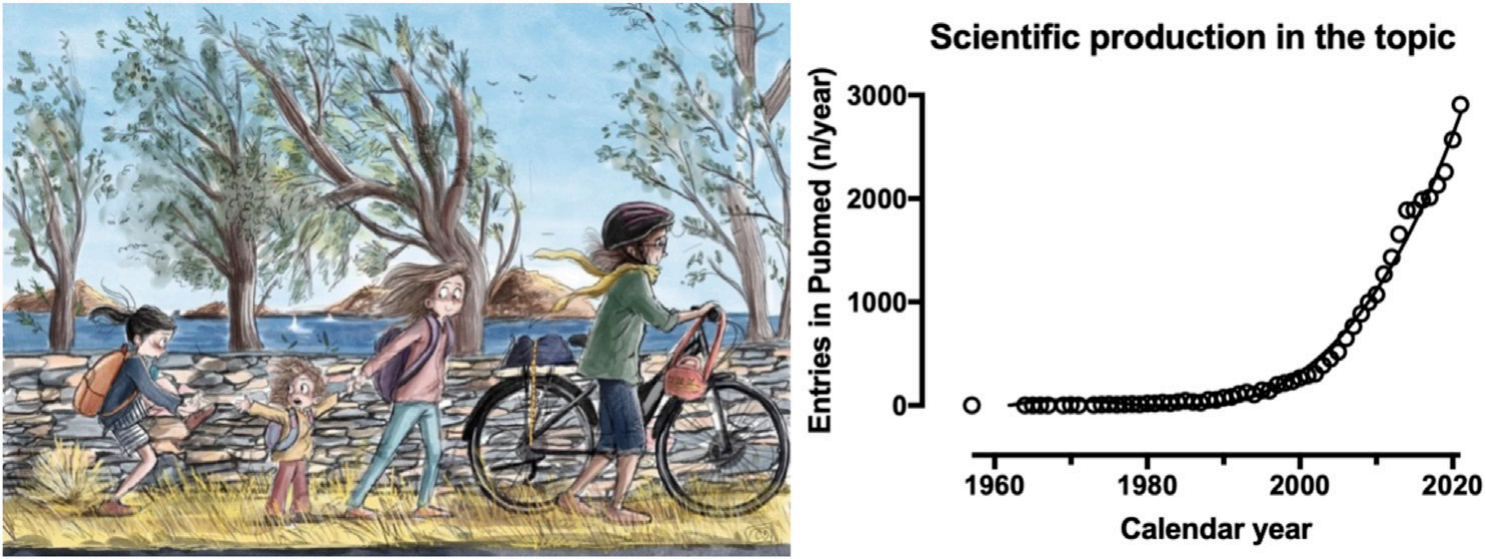
Entries in Pubmed using the syntax [“physical activity” AND (sex OR gender)] according to search conducted on 30 June 2022.

A search of the Pubmed database using the syntax [“physical activity” AND (gender OR sex)] showed an increasing number of entries across calendar years (Figure 1). The first entry was in 1957, there were 18 entries in 1980, 73 in 1990, 266 in 2000, 1,071 in 2010 and 2,908 in 2021. This trend reflects an ongoing scientific interest in the study of differences in physical activity between women and men.

The present Research Topic aimed to address the above mentioned research questions including seven scientific papers. Slater et al. examined the association between PA, body composition and biomarkers of metabolic health in Pacific and New Zealand European women who are known to have different metabolic disease risks. They observed that increases in total physical activity and mean moderate-to-vigorous physical activity are associated with lower fasting plasma insulin, thus indicating a reduction in metabolic disease risk. In another study, Frandsen et al. studied metabolic responses to fasting and exercise. They showed that peak fat oxidation increased with fasting and repeated exercise in trained women, but the relative peak fat oxidation was similar in young trained men and women, despite major differences in plasma lipid concentrations during graded exercise.

Xu et al. reviewed the effects of physical exercise, antioxidative supplementation, and their combination on the dynamic balance between oxidation and anti-oxidation in different subgroups of healthy adults of both sexes. They concluded that keeping a regular physical exercise routine and gradually increasing its intensity according to the individual’s daily life activity might be a better choice to maintain and enhancing the body’s antioxidation potential, only using anti-oxidative supplementation is not recommended. In addition, Espinosa-Ramirez et al. compared oxygen saturation in respiratory (SmO2-m.intercostales) and locomotor muscles (SmO2-m.vastus lateralis) while performing physical exercise in women and men. They highlighted that during an incremental physical exercise, women experienced a greater cost of breathing, reflected by greater deoxygenation of the respiratory muscles, whereas men had a higher peripheral load, indicated by greater deoxygenation of the locomotor muscles.

Lanfranchi et al. tested whether gender interferes with the spleen perfusion and its response to exercise. They concluded that exercise interference on spleen perfusion can be detected during myocardial perfusion imaging and this effect is dependent upon gender and ischemia confirming the high sensitivity of this organ to sympathetic nervous system activation. Furthermore, Rodziewicz-Flis et al. focused on the relationship of 25(OH)D concentration with physical training responses and physical performance, oxidative stress markers, inflammation, and bone metabolism in older women. They concluded that vitamin D concentration among older women is associated with physical performance, fall risk, inflammation, and bone metabolism markers, and 12 weeks of training improved physical performance and antioxidant protection, regardless of baseline vitamin D concentration.

Finally, Militello et al. studied sex-related sports adaptation proteins in female basketball players and male basketball players using proteomics approach on plasma samples withdrawn from athletes during in-season training period but far from a competition. They identify in female athletes a reduction in proteins related to transcription regulation, most of these modulate chronic inflammation confirming the anti-inflammatory effect of regular training in female muscle metabolism. A decrease in Transthyretin involved in muscle homeostasis and regeneration and Dermcidin a stress-induced myokine linked to inflammatory was observed in both female and male athletes. At this point, we wish to thank all authors submitting their work to this Research Topic, reviewers, associate editors and the staff of Frontiers. Without their collaborating effort, this Research Topic could not be realized. This Research Topic intended to draw attention to the gap in gender-based studies focusing on the response to exercise, also critical to better understand the basis for the sex-related physiopathology and epidemiological differences in the onset and development of chronic diseases (e.g., cardiometabolic and neurodegenerative diseases and cancer).

Actually there are still many unsolved issues in the gender-related responses to exercise. Further deepening of such aspects can be the basis for future studies explaining the different physiological characteristics as well as pathogenesis and observed epidemiological differences of chronic degenerative diseases (cardiovascular and neurodegenerative diseases and cancer) in males and females. This knowledge may help to better target interventions preserving health and fitness and managing diseases from a gender point of view. Despite the increasing studies about woman and man in physical activity, to date we know very little about sex-related differences and similarities in exercise; in fact women are still under-represented compared to men in sport medicine research and further studies should be emphasized to fully understand the female physiology and metabolism during exercise. Moreover, relevant issues are still open in this Research Topic including 1) understanding the molecular mechanism of different energetic substrates utilization in woman and man during exercise and recovery; 2) evaluating the impact of nutrient supplementation in woman and man; 3) understanding the sex-related hormone signalling in exercise; and 4) assessing the sex-related antioxidant mechanism involved in exercise.
